# Optimal loading dose of meropenem before continuous infusion in critically ill patients: a simulation study

**DOI:** 10.1038/s41598-021-96744-3

**Published:** 2021-08-26

**Authors:** Uwe Liebchen, Hanna Salletmeier, Simon Kallee, Christina Scharf, Lucas Huebner, Alexandra Weber, Michael Zoller

**Affiliations:** 1 Department of Anesthesiology, University Hospital, LMU Munich, Marchioninistrasse 15, 81377 Munich, Germany; 2 Department of Hospital Pharmacy, University Hospital, LMU Munich, Marchioninistr. 15, 81377 Munich, Germany

**Keywords:** Antibiotics, Bacterial infection

## Abstract

The aim of this study was to investigate optimal loading doses prior to continuous infusion of meropenem in critically ill patients. A previously published and successfully evaluated pharmacokinetic model of critically ill patients was used for stochastic simulations of virtual patients. Maintenance doses administered as continuous infusion of 1.5–6 g/24 h with preceding loading doses (administered as 30 min infusion) of 0.15–2 g were investigated. In addition to the examination of the influence of individual covariates, a best-case and worst-case scenario were simulated. Dosing regimens were considered adequate if the 5th percentile of the concentration–time profile did not drop at any time below four times the S/I breakpoint (= 2 mg/L) of *Pseudomonas aeruginosa* according to the EUCAST definition. Low albumin concentrations, high body weight and high creatinine clearances increased the required loading dose. A maximum loading dose of 0.33 g resulted in sufficient plasma concentrations when only one covariate showed extreme values. If all three covariates showed extreme values (= worst-case scenario), a loading dose of 0.5 g was necessary. Higher loading doses did not lead to further improvements of target attainment. We recommend the administration of a loading dose of 0.5 g meropenem over 30 min immediately followed by continuous infusion.

## Introduction

Meropenem is regularly used to treat life-threatening infections in critically ill patients, as it covers a broad spectrum of pathogens and shows a bactericidal mechanism of action^1^. Prompt initiation of an effective therapy is imperative, as each hour of delay increases mortality by approximately 7%^2^. In common with all beta-lactams, meropenem exerts its activity via a time-dependent mechanism, i.e. the relevant pharmacokinetic/pharmacodynamic (PK/PD) index is the time of the free concentration exceeding the minimum inhibitory concentration of the pathogen (*f*T > MIC)^3^. Continuous infusion offers theoretical advantages to improve this index: Roberts et al. detected in a meta-analysis a lower in-hospital mortality with continuous infusion compared to intermittent infusion^4^. However, administering meropenem as a continuous infusion and starting with the maintenance rate would lead to a period below the MIC in the increasing part of the concentration-time profile. This should be avoided especially at the beginning of a course of treatment, when adequate antibiotic exposure is key^2,5^. In order to raise the concentration as quickly as possible above the MIC, the administration of an initially higher dose, i.e. a “loading dose”, is recommended^6^. However, according to previously published guidelines on the optimization of the treatment with beta lactam antibiotics in critically ill patients, there is currently no consensus on the amount of a loading dose which should be used in terms of rational antibiotic therapy for meropenem with subsequent continuous infusion^6^.

Therefore, this study aimed to identify the optimal loading dose when meropenem is subsequently administered as continuous infusion in critically ill patients. For this purpose, a simulation study was conducted based on data of a prospective PK study.

## Material and methods

### Pharmacokinetic model

A previously published two compartment model with first-order elimination of critically ill patients was employed for simulation^7^. This model was recently successfully evaluated and revealed a neglectable bias^8^. Briefly, this model is based on a prospective observational study in a population of 48 critically ill patients with severe infections and dense PK sampling (n = 1376) over 4 days. All patients were administered meropenem standard doses three times daily as a short infusion over 30 min. For further information regarding the original data, please refer to Ehmann et al.^7^. Based on this study, a nonlinear-mixed effect model was developed including three covariates: creatinine clearance (CLCR) according to Cockcroft and Gault^9^ as a covariate on meropenem clearance, total body weight on the central volume of distribution and serum albumin concentration on the peripheral volume of distribution, implemented as piecewise linear, power and linear relationship, respectively^7^. Alterations in albumin concentrations and body weight influence the volume of distribution of meropenem according to the employed PK model, i.e. low albumin concentrations lead to an increased peripheral volume of distribution, while a higher body weight leads to a higher central volume of distribution.

### Pharmacokinetic/pharmacodynamic target

A PK/PD target of 100% T > 4xMIC was selected, which has recently been associated with maximizing therapeutic effect and minimizing the spread of resistance of beta-lactams^6^. Due to the negligible protein binding of meropenem, only total meropenem concentrations were considered^10,11^. Given the non-achievability of target attainment in the very first period of the administration of the loading dose (increasing part of the concentration-time profile during the infusion of the loading dose), we ignored the first 30 min of infusion. Since there is no MIC available at the start of therapy in the case of empirical therapy, we used the unspecific S/I breakpoint (= 2 mg/L) for *Pseudomonas aeruginosa* provided by the EUCAST^12^. Dosing regimens were considered adequate if the 5^th^ percentile of the concentration-time profile did not drop below 8 mg/L at any time after administration of the loading dose, i.e. the PK/PD target of 100% T>4xMIC was achieved.

### Simulations

All simulations were performed using the R-package mrgsolve (R version 4.02, CRAN.R-project.org). Three daily doses (1.5 g, 3 g, 6 g) were simulated stochastically (n_simulations_ = 1000). Daily maintenance doses of 1.5 g were administered for patients with CLCR ≤ 30 mL/min, 3 g for CLCR > 30 mL/min and ≤ 80 ml/min and 6 g for patients with CLCR > 80 mL/min in order to achieve sufficient steady state concentrations (based on^7^).

Every daily dose was combined with six different loading doses (0.15 g, 0.25 g, 0.33 g, 0.5 g, 1 g, 2 g), with the loading dose being administered over 30 min and the daily dose administered immediately thereafter as a continuous infusion. Four different scenarios were investigated:

Scenario 1: simulation of patients with varying renal function (CLCR 0–160 mL/min, step-size: 10 mL/min), albumin and body weight were fixed (2.8 g/dL, 70 kg).

Scenario 2: simulation of patients with varying albumin levels (1–4 g/dL, step-size: 0.25 g/dL), CLCR and body weight were fixed (80 mL/min, 70 kg).

Scenario 3: simulation of patients with varying body weight (50–150 kg, step-size: 10 kg), CLCR and albumin were fixed (80 mL/min, 2.8 g/dL).

Scenario 4: simulation of a best- and worst-case scenario, i.e. in the worst case scenario a patient was simulated with CLCR of 160 mL/min, albumin of 1 g/dL and body weight of 150 kg (daily dose 6 g). In the best-case scenario a patient was simulated with CLCR of 0 mL/min, albumin of 4 g/dL and body weight of 50 kg (daily dose 1.5 g).

## Results

### Influence of creatinine clearance

**Figure 1:**
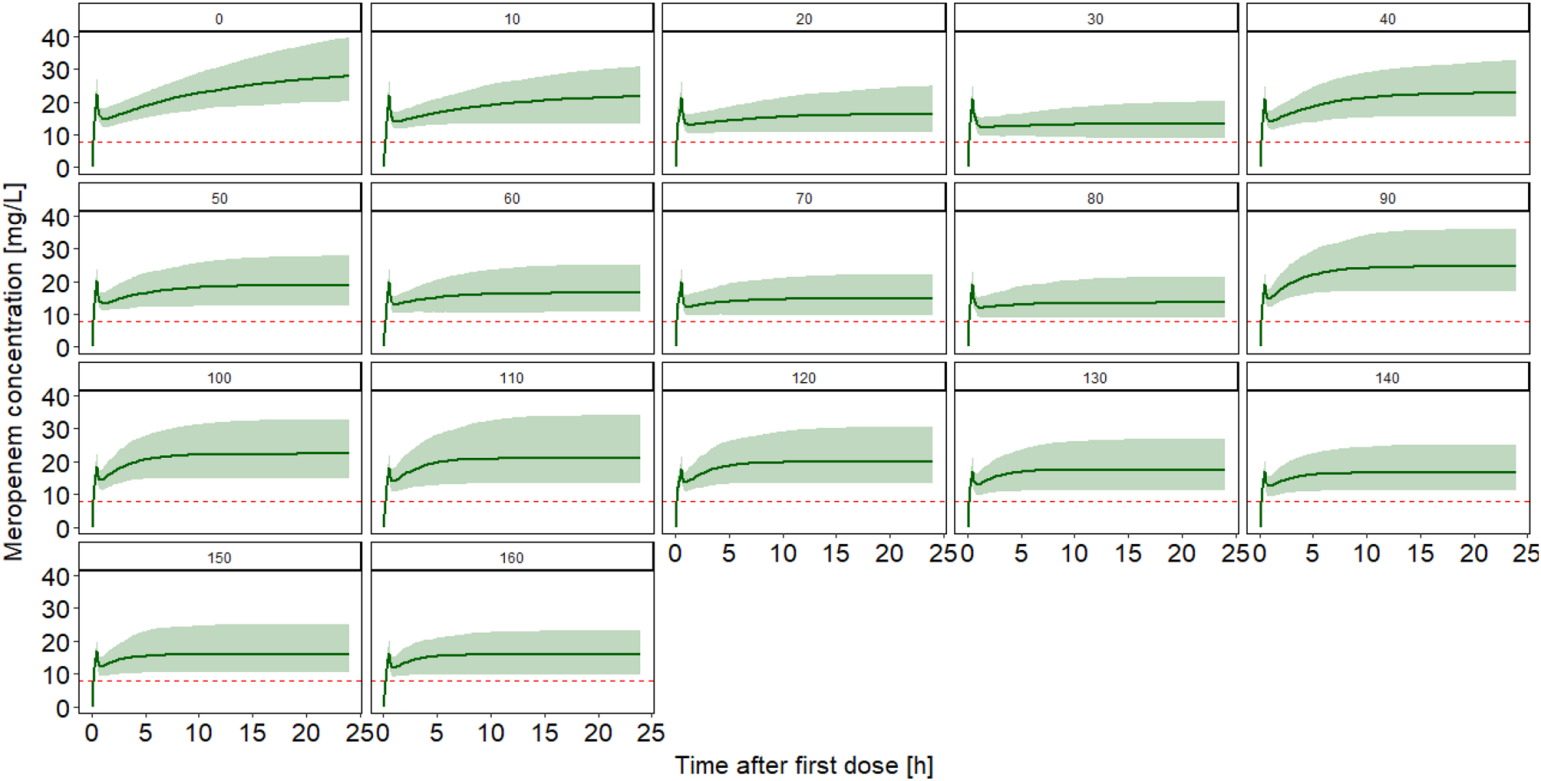
Predicted plasma concentration–time profile in patients with varying kidney function and a loading dose of 0.33 g. Patients with varying renal function (creatinine clearance according to Cockcroft and Gault^9^ 0–160 mL/min, step-size: 10 mL/min, respective numbers in the plots indicate the individual creatinine clearance), albumin was fixed to 2.8 g/dL, body weight was fixed to 70 kg. Daily doses of 1.5 g were administered for patients with creatinine clearances of 0–30 mL/min, 3 g for creatinine clearances 40–80 mL/min and 6 g for patients with creatinine clearances 90–160 mL/min. Line: median concentration, Shaded area: 90% prediction interval, Horizontal dotted line: 4 × S/I breakpoint of Pseudomonas aeruginosa according to^12^.

**Figure 2:**
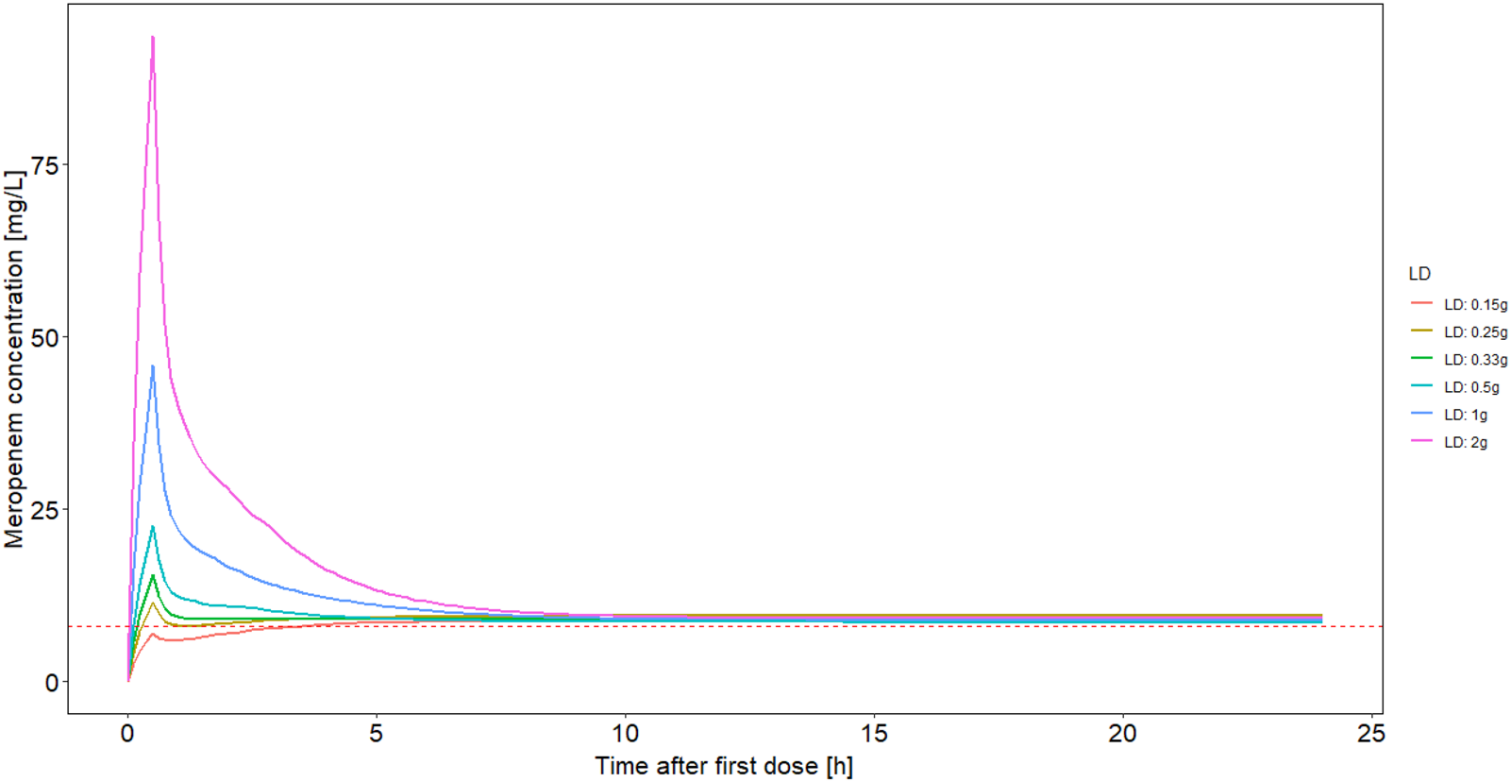
Predicted plasma concentration–time profile (5^th^ percentile) in a patient with a creatinine clearance of 80 mL/min and varying loading doses. Albumin was fixed to 2.8 g/dL, body weight was fixed to 70 kg. A daily dose of 3 g was administered. Lines: 5th percentile of the concentration time profile, Horizontal dotted line: 4 × S/I breakpoint of Pseudomonas aeruginosa according to^12^. LD: loading dose.

First, the influence of the renal function was evaluated. A loading dose of 0.15 g revealed insufficient minimum concentrations for all simulated regimens and CLCR values and led to a delay until adequate concentrations were achieved. A loading dose of 0.25 g was not sufficient for patients with all examined values of CLCR, as the minimum value of the 5th percentile of the concentration-time profile was below the 8 mg/L limit for very high CLCR values (e.g. CLCR 160 mL/min: 7.93 mg/L). A loading dose of 0.33 g revealed adequate concentrations for all patients by being clearly above the threshold of 8 mg/L for 100% of the time (lowest 5th percentile: 9.0 mg/L for patients with CLCR 160 mL/min). Figure 1 illustrates the influence of the renal function. Figure 2 shows the influence of different loading doses graphically.

### Influence of albumin-concentrations

Higher loading doses were needed in patients with low albumin concentrations compared to patients with high albumin concentrations. As described in Scenario 1 (patients with varying kidney function), a loading dose of 0.15 g resulted in a delayed achievement of adequate concentrations for all examined albumin-concentrations. Using a loading dose of 0.25 g, sufficient plasma concentrations could be achieved in some, but not all cases immediately after infusion of the loading dose. For example, a patient with a loading dose of 0.25 g, a CLCR of 80 mL, a body weight of 70 kg, a daily dose of 3 g and an albumin concentration of 1 g/dL achieved a median minimum concentration of 9.1 mg/L (5^th^ percentile: 7.40 mg/L). In contrast, a patient with the same covariates but an albumin concentration of 4 g/dL achieved median minimum concentrations of 11.5 mg/L (5^th^ percentile: 9.5 mg/L). However, with a loading dose of 0.33 g, all patients showed plasma concentrations clearly above the threshold of 8 mg/L for 100% of the time.

### Influence of body weight

Similar to the albumin-scenario, body weight influenced the required loading dose; higher loading doses are needed in patients with higher body weight. A loading dose of 0.15 g led to insufficient post-loading dose plasma concentrations. Sufficient concentrations could partly be achieved with a loading dose of 0.25 g: a patient with a loading dose of 0.25 g, a CLCR of 80 mL/min, a body weight of 150 kg, a daily dose of 3g and an albumin concentration of 2.8 g/dL achieved only a minimum median concentration of 8.8 mg/L (5^th^ percentile: 6.9 mg/L), while a patient with the same CLCR and albumin concentration but a body weight of 50 kg achieved concentrations of 10.8 mg/L (5^th^ percentile: 8.5 mg/L). With a loading dose of 0.33 g, all patients showed concentrations clearly above the threshold of 8 mg/L for 100% of the time.

### Best-case/worst-case scenario

**Figure 3:**
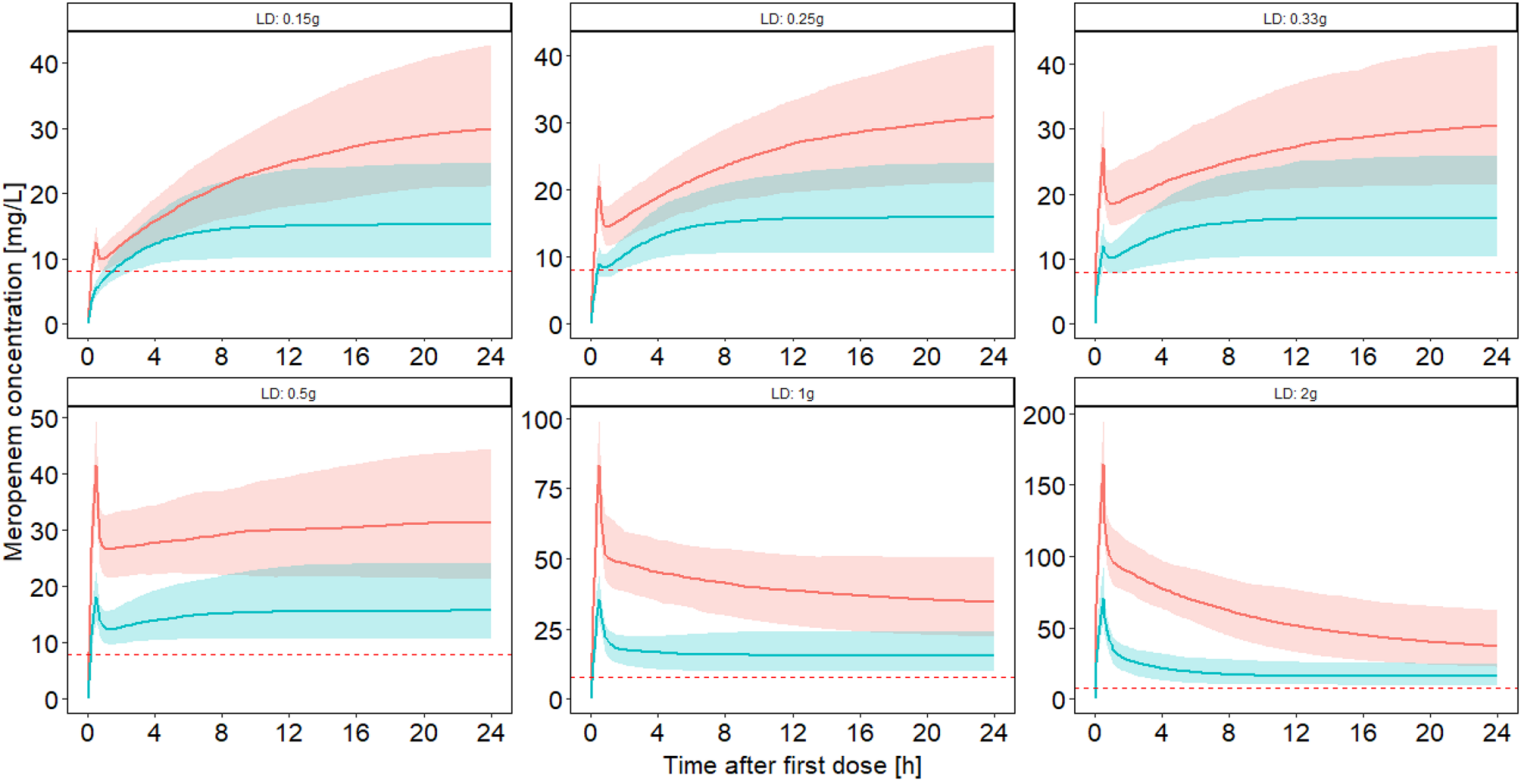
Predicted plasma concentration–time profile in a best- and worst-case scenario with six different loading doses. in the worst case scenario (blue line = median concentration, blue area = 90% prediction interval) the profile of a patient with a creatinine clearance according to Cockcroft and Gault^9^ of 160 mL/min, albumin concentration of 1 g/dL and body weight of 150 kg (maintenance dose: 6 g/24 h) was simulated. In the best-case scenario (red line = median concentration, red area = 90% prediction interval) the profile of a patient with creatinine clearance of 0 mL/min, albumin concentration of 4 g/dL and body weight of 50 kg (maintenance dose: 1.5 g/24 h) was simulated. Horizontal dotted line: 4 × S/I breakpoint of Pseudomonas aeruginosa according to^12^. LD: loading dose.

Without a loading dose, it would take 3.4 and 2.6 h for the 5^th^ percentile to reach the target concentration in the best- and worst-case scenarios, respectively. The accelerated achievement of the target concentration in the worst-case scenario can be explained by the higher infusion rate (6 g/24 h vs. 1.5 g/24 h). All loading doses of 0.25 g or higher led to the achievement of the PK/PD target in the best-case scenario. In contrast, in the worst-case scenario, a loading dose of 0.25 g showed insufficient concentrations in the beginning: the 5th percentile of the minimum of the concentration-time profile fell clearly below the target concentration of 8 mg/L (median: 8.66 mg/L, 5^th^ percentile: 6.6 mg/L). Also, a loading dose of 0.33 g showed a short period when the 5^th^ percentile of the concentration-time profile dropped below the target range of 8 mg/L (7.8 mg/L). Loading doses greater or equal to 0.5 g revealed no drops of the concentrations at any timepoint below 8 mg/L and were therefore considered adequate in the worst-case scenario. Figure 3 illustrates the best- and worst-case scenario.

## Discussion

The administration of a loading dose before the initiation of continuous infusion is generally accepted and recommended for beta-lactams^6^. In the majority of studies investigating continuous infusion loading doses were administered, including the large multicenter studies BLISS and BLING II^6,13,14^. This practice is supported by a significant mortality reduction when loading doses are administered compared to no loading doses according to a large meta-analysis by Vardakas et al.^15^. However, in previous studies with continuous infusion of meropenem, heterogeneous loading doses of 0.5–2 g were administered, indicating that reliable recommendations on the optimal dose are missing^7,16–18^. Guilhaumou et al. recommended in the absence of consensus in their guideline on optimizing beta-lactam therapy in critically ill patients administering a loading dose identical to that used in the case of discontinuous administration^6^.

In the present study, we extensively analyzed which amount of a loading dose should be administered to achieve sufficient concentrations at the onset of continuous infusion therapy for meropenem. For this purpose, we followed a simulation approach based on a previously published PK model. The employed model was developed based on densely collected samples and a high number of heterogenous critically ill patients^7^. The advantage of a simulation-based approach compared to a prospective study is, besides the lower costs, the possibility to investigate an unlimited number of possible combinations of loading doses and infusion rates. Furthermore, especially when investigating a short time interval of drugs with a short half-life (here: time when loading dose of meropenem is administered), small protocol deviations (e.g. sample collection 5 min to late) would lead to massive inaccuracies of the measured concentrations^19^. It should be noted as a strength of this study that the model employed can be considered valid since the model was evaluated externally showing negligible bias^8^. The evaluation of the PK model revealed a negligibly small underestimation of the measured concentrations (− 0.84 mg/L). In the context of this simulation study, this would mean that the actual concentrations would tend to be slightly higher than the predicted concentrations indicating a further safety margin (towards subtherapeutic exposure). A previous study by Delattre et al. also used a simulation-based approach to identify the optimal loading dose of four beta-lactams including meropenem^20^. Unfortunately, this study did not investigate which loading doses should be administered if continuous infusion is immediately started after the loading dose (only intermittent dosing regimens were investigated). The authors suggested a loading dose of 2 g meropenem, administered over 30 min, with the next dose 6 h later. Therefore, the loading dose proposed in the study by Delattre et al. is significantly higher than the one identified in our study. However, it should be assessed in the circumstance that concentrations continue to drop until the next dose is administered 6 hours later, which is not the case if the infusion is administered at the maintenance rate immediately after the end of the loading dose.

Maintenance doses were adjusted for renal function in our study. This approach corresponded in all cases with steady state concentrations in the therapeutic range and most closely reflects actual practice in the administration of meropenem in critically ill patients. While CLCR is decisive for the required continuous infusion rate, albumin and body weight (as covariates on the volumes of distribution) are relevant for the loading dose. A low loading dose of 0.33 g was sufficient when only one of the three investigated covariates (CLCR, albumin, body weight) showed extreme values. However, in a worst-case scenario, a loading dose of 0.5 g resulted in adequate plasma concentrations. This loading dose of 0.5 g was previously described as adequate prior to continuous infusion in simulations, is commercially available and was used in a clinical context^21^. Toxic concentrations are not expectable from this loading dose. Higher loading doses do not promise any further advantage in target attainment, but potentially might lead to toxic effects. Neurotoxicity and nephrotoxicity have been reported with high trough levels of meropenem during intermittent infusion, however, no data on toxic peak levels are available to date^22^.

This study examines purely the relationship between the attainment of a defined PK/PD target and the amount of a loading dose. However, the investigation of clinical outcomes related to PK/PD targets should be further addressed in future studies.

## Conclusions

This is the first study that investigated optimal loading doses with subsequent continuous infusion of meropenem in critically ill patients. Administration of a loading dose of 0.5 g over 30 min immediately followed by continuous infusion promises instantaneous achievement of sufficient target concentrations. Therefore, we recommend the application of 0.5 g meropenem as a loading dose for all critically ill patients. Higher loading doses do not lead to further improvements in target attainment.

## Data Availability

Code is available via Email request to uwe.liebchen@med.uni-muenchen.de.

## Abbreviations

S/I: Susceptible/susceptible with increased exposure
EUCAST: European Committee on Antimicrobial Susceptibility Testing
PK/PD: Pharmacokinetic/Pharmacodynamic
fT > MIC: Time of the free drug above the minimal inhibitory concentration
CLCR: Clearance creatinine according to Cockcroft and Gault
LD: Loading dose
